# Intracranial direct electrical mapping reveals the functional architecture of the human basal ganglia

**DOI:** 10.1038/s42003-022-04084-3

**Published:** 2022-10-23

**Authors:** Lei Qi, Cuiping Xu, Xueyuan Wang, Jialin Du, Quansheng He, Di Wu, Xiaopeng Wang, Guangyuan Jin, Qiao Wang, Jia Chen, Di Wang, Huaqiang Zhang, Xiaohua Zhang, Penghu Wei, Yongzhi Shan, Zaixu Cui, Yuping Wang, Yousheng Shu, Guoguang Zhao, Tao Yu, Liankun Ren

**Affiliations:** 1 Department of Neurology, Xuanwu Hospital, Clinical Center for Epilepsy, Capital Medical University, Beijing, China; 2 National Center for Neurological Disorders, Beijing, China; 3 Department of Functional Neurosurgery, Beijing Institute of Functional Neurosurgery, Xuanwu Hospital, Clinical Center for Epilepsy, Capital Medical University, Beijing, China; 4 Department of Pharmacy Phase I Clinical Trial Center, Xuanwu Hospital, Clinical Center for Epilepsy, Capital Medical University, Beijing, China; 5 Department of Neurology, Huashan Hospital, State Key Laboratory of Medical Neurobiology, Institute for Translational Brain Research, MOE Frontiers Center for Brain Science, Fudan University, Shanghai, China; 6 School of Engineering Medicine, Beihang University, Beijing, China; 7 Department of Neurosurgery, Xuanwu Hospital, Clinical Center for Epilepsy, Capital Medical University, Beijing, China; 8 Chinese Institute for Brain Research, Beijing, China

**Keywords:** Epilepsy, Parkinson's disease

## Abstract

The basal ganglia play a key role in integrating a variety of human behaviors through the cortico–basal ganglia–thalamo–cortical loops. Accordingly, basal ganglia disturbances are implicated in a broad range of debilitating neuropsychiatric disorders. Despite accumulating knowledge of the basal ganglia functional organization, the neural substrates and circuitry subserving functions have not been directly mapped in humans. By direct electrical stimulation of distinct basal ganglia regions in 35 refractory epilepsy patients undergoing stereoelectroencephalography recordings, we here offer currently the most complete overview of basal ganglia functional characterization, extending not only to the expected sensorimotor responses, but also to vestibular sensations, autonomic responses, cognitive and multimodal effects. Specifically, some locations identified responses weren’t predicted by the model derived from large-scale meta-analyses. Our work may mark an important step toward understanding the functional architecture of the human basal ganglia and provide mechanistic explanations of non-motor symptoms in brain circuit disorders.

## Abstract (web summary)

Direct electrical stimulation of the basal ganglia using implanted SEEG electrodes produced a variety of motor and non-motor effects in human participants, providing insight into the functional architecture of this key brain region.

## Introduction

A fine-grained characterization of human behaviors at the level of cortico-subcortical circuitry remains one of the major goals of neuroscientific research. The basal ganglia (BG), encompassing the striatum, globus pallidus, subthalamic nucleus (STN), and substantia nigra, are an exquisite, complex system of interconnected subcortical nuclei situated in the center of the brain^1,2^. These nuclei receive information from the cortex and then project back to cortical structures through the thalamus in circuits known as the cortico-BG-thalamo-cortical loops; as part of these loops, the BG are crucial hubs in the integration of various human behaviors^3–5^. Accordingly, functional and anatomical aberrations of the BG have been implicated in a broad clinical spectrum of debilitating neuropsychiatric disorders with prominent motor features and myriads of non-motor symptoms; these conditions, known as brain circuit disorders, affect a vast number of individuals in the population, and the resulting burden of disease is rapidly increasing worldwide^6,7^.

Probing and identifying the processes of physiological functions in the human BG has both scientific and clinical relevance. Initial knowledge of BG function stemmed from causal inference based on the clinical manifestations of patients with focal BG lesions and neurodegenerative diseases. Based on physiological and anatomical observations in nonhuman primates, the milestone model of five functionally segregated and largely closed circuits proposed by Alexander et al.^3,8^ in the 1980s provided a neurobiological basis for BG function. More recently, with the advent of new methodologies in animal studies and the surge of neuroimaging research, a set of novel intrinsic and extrinsic BG pathways has been uncovered^9–11^. With the proliferation of knowledge regarding BG function, the BG is now known to be essential for the learning, coordination, and execution of sequenced motor actions via the direct, indirect, and hyperdirect pathways. Moreover, substantial evidence has implicated the BG in a growing list of complex cognitive and affective processes^12–14^. Indeed, the understanding of BG functional organization has accelerated the neuromodulation interventions for brain circuit disorders over the past few decades. At present, deep brain stimulation (DBS) is a well-established and effective treatment modality to alleviate motor symptoms of Parkinson’s disease (PD) and other movement disorders^15–17^. The effectiveness of DBS, however, has not been well replicated for all manifestations of movement disorders; non-motor symptoms and signs have especially low response rates^18,19^, reflecting the fact that some aspects of BG function are still not fully understood.

Classically, direct electrical stimulation (DES) has long been recognized as a standard clinical approach to exploring the primary and high-order functions in the brain^20,21^. Notably, microstimulation of the BG has been conducted in animal experiments and clinical DBS procedures. Stimulation of the BG in animal experimentation has yielded important information^22^. Nevertheless, the subjective effects and high-order functions that account for human highly orchestrated behaviors are still an open question. In clinical DBS procedures for patients suffering from movement disorders, DES mapping serves to refine the selection and boundaries of targets by evoked sensorimotor effects; meanwhile, evoked non-motor responses have been sporadically reported as well. However, the information acquired through DES with the DBS lead is largely restricted because DBS leads are usually directed toward the STN and internal globus pallidus (GPi) and follow similar trajectories across patients. Importantly, almost all prior clinical DBS studies relied on patients suffering from BG-related diseases, and the exact physiological nature of BG function is still somewhat ambiguous.

Here, we address this gap by characterizing the acute motor and non-motor responses elicited using DES of the BG in a group of refractory epilepsy patients who underwent clinically indicated intracranial recordings for chronic stereoelectroencephalography (SEEG) monitoring. For each patient in this group, at least one electrode targeting the anterior nucleus of the thalamus (ANT) or STN was used to probe the cortico-subcortical epileptic network and refine potential DBS targets for modulating epileptic activity, which has been reported in our previous studies^23,24^. Importantly, the personalized SEEG trajectories passed through distinct regions of the BG among individuals, with relatively broad spatial sampling in the group. In particular, the use of DES to evaluate these individuals without functional deficits in the BG offered a unique chance to directly map the physiological function of the BG, including the sensorimotor responses, vestibular sensations, autonomic responses, cognitive and multimodal effects, which are involved in human complex behaviors.

## Results

### The spatial distribution of electrode contacts within the BG

**Fig. 1:**
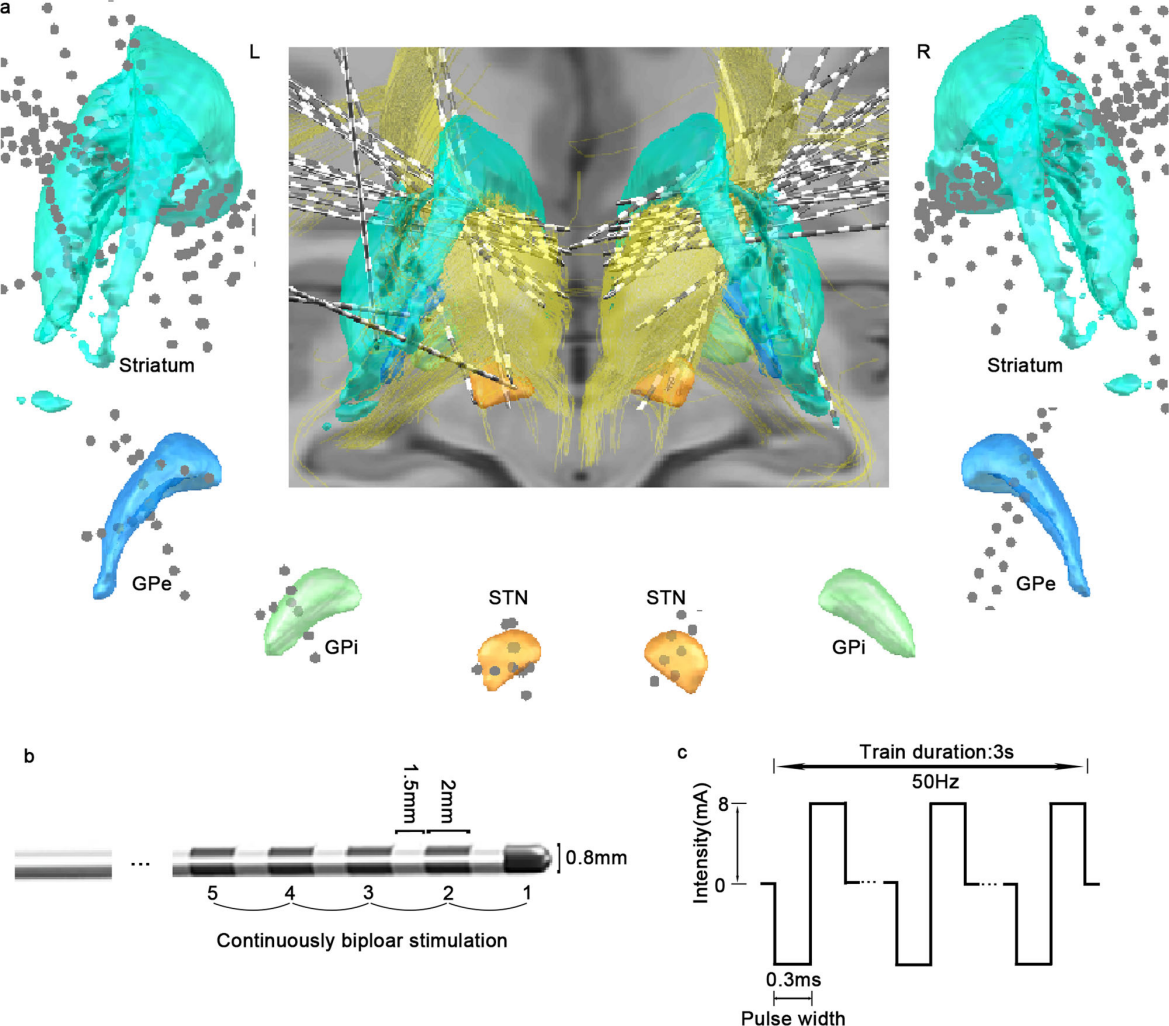
Illustration of grouped electrodes passing through the BG in MNI space (ICBM 2009b), electrode configuration, and stimulus parameters. **a** 3D reconstruction of the multisite SEEG electrodes (*n* = 42) passing through the BG (striatum in turquoise, GPe in blue, GPi in green, and STN in orange) as defined by the DISTAL Atlas, together with the anterior limb of the internal capsule (yellow). The gray dots represent the sites of the SEEG electrodes. The electrodes passing through each BG subnucleus are illustrated. **b** SEEG electrode configuration used for stimulation. **c** Graphic illustration of the stimulus parameters using a rectangular biphasic pulse. MNI Montreal Neurological Institute, L left, R right, SEEG stereoelectroencephalography, BG basal ganglia, GPe external globus pallidus, GPi internal globus pallidus, STN subthalamic nucleus.

We determined the locations of the electrodes in the BG subnuclei based on the DISTAL and Brainnetome atlases (Supplementary Figure 1)^25,26^. In total, 42 out of 297 implanted multisite SEEG electrodes (8–20 contacts, 2 mm in length, 1.5 mm apart) from 39 patients were identified as passing through diverse regions of the BG (Supplementary Table 1). The spatial distribution of all the electrodes within the BG subnuclei is displayed in the Montreal Neurological Institute (MNI) standard space in Fig. 1a. To avoid the potential effects of stimulating the adjacent fibers, we initially took care to exclude the patients whose contacts appeared to cross the boundary of the BG by visual inspection. Ultimately, 35 patients were included.

After visual inspection, 80 electrode contact sites were detected strictly within the BG. Among them, one site exhibiting after discharges indicating the high excitability of the tissue was excluded from further analysis. Finally, 79 sites from 35 patients have included: 63 sites in the striatum (13 sites in dorsal caudate (dCa); 18 sites in dorsolateral putamen (dlPu); 32 sites in ventromedial putamen (vmPu)), 7 sites in the external globus pallidus (GPe), 2 sites in the GPi, and 7 sites in the STN (Supplementary Data 1).

### Elicitation of responses: current intensities delivered to the BG

**Fig. 2:**
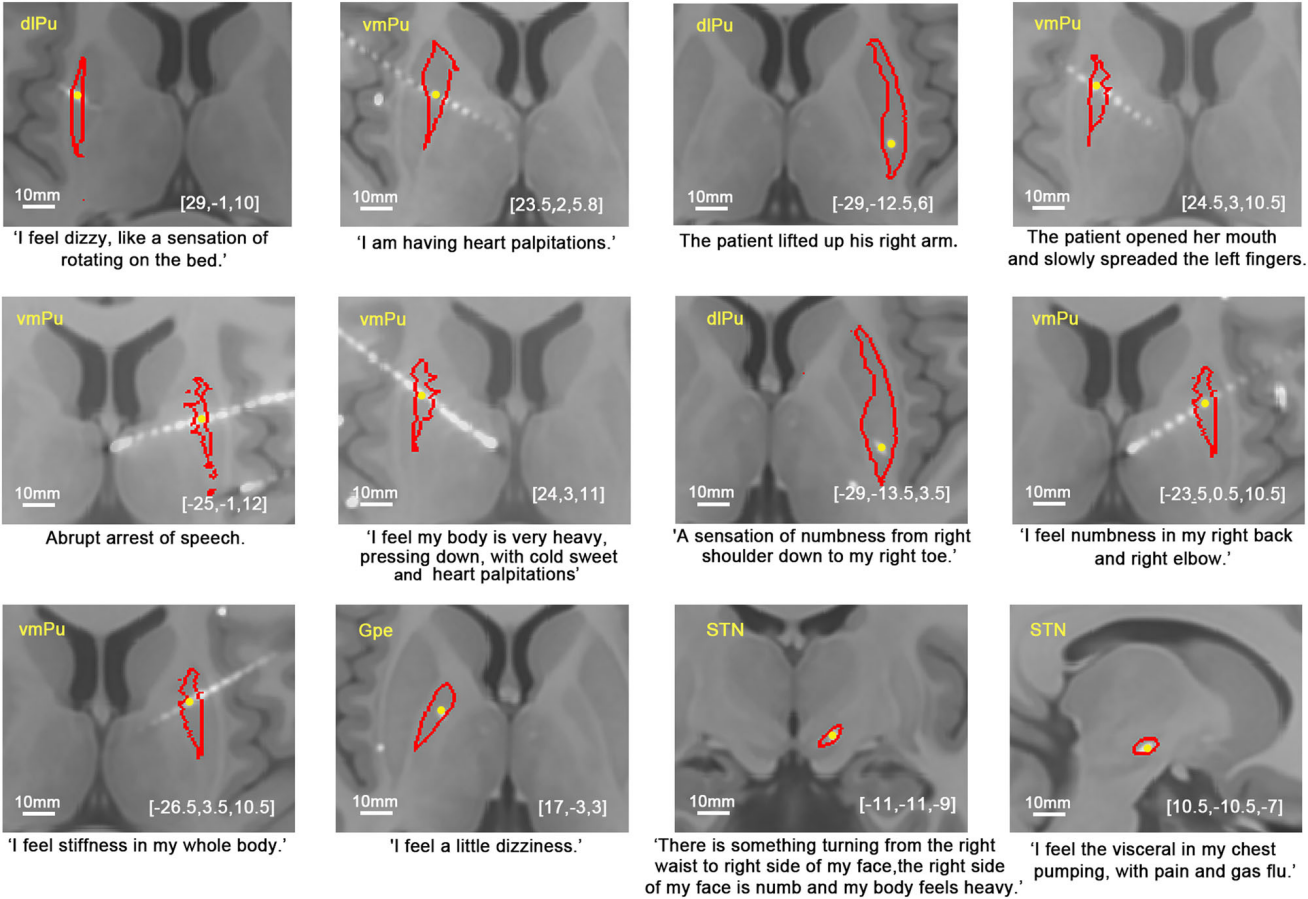
Examples of the diverse motor and non-motor responses arising from DES of the BG. These panels show examples of individual contact sites in various BG subnuclei (red contour line) in MNI space and detailed reports and/or objective responses from patients during the stimulation of the corresponding sites (yellow dots). The white line of the scale bar indicates 10 mm distance. DES direct electrical stimulation, vmPu ventromedial putamen, dlPu dorsolateral putamen.

After the SEEG implantation operation, the patients returned from the operating room to the ward and underwent 7–14 days of SEEG monitoring to capture their habitual clinical seizures. DES was conducted during the chronic SEEG monitoring. The SEEG DES process for each patient took at least one to two days depending on the number of electrode contacts and the state of the patient. On bipolar DES (illustrated in Fig. 1b, c) of the BG subnuclei, 54 sites were identified at which DES reproducibly elicited clinical responses/behaviors. The acute elicited responses were highly heterogeneous and included limb movements, dystonic gestures, eye deviations, somatosensory responses, and a diverse array of non-motor behaviors. Full details of the patient reports and objective observations are presented in Supplementary Data 1; example responses and corresponding coordinates of the activated sites are shown in Fig. 2.

**Fig. 3:**
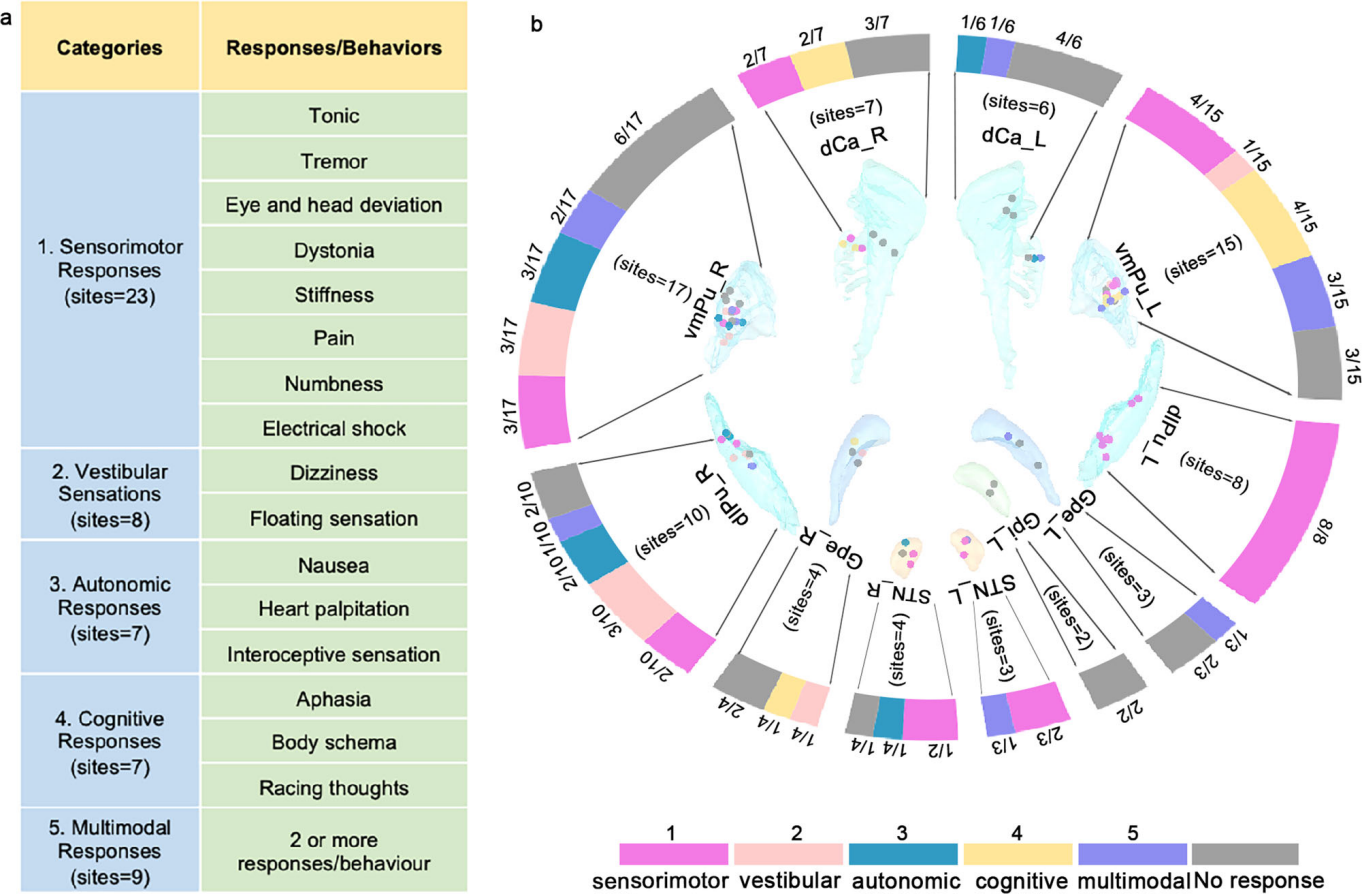
Direct electrical stimulation elicited diverse responses. **a** Elicited responses/behaviors were classified into five categories based on their properties. **b** The spatial distribution of contacts and the rate of different responses/behaviors elicited within the BG subnucleus. The response categories are color-coded.

Based on the characteristic profiles and the possible function of BG, the elicited responses were here classified into five categories: (1) sensorimotor responses, which were subdivided into the motor and somatosensory responses; (2) vestibular sensations; (3) autonomic responses; (4) cognitive responses; and (5) multimodal responses (the co-occurrence of responses from more than one other category; Fig. 3a). The intensity of the stimulation current was 3.62 ± 2.06 mA for sensorimotor responses, 3.88 ± 2.88 mA for vestibular sensations, 2.86 ± 1.46 mA for autonomic responses, 5.64 ± 1.27 mA for cognitive responses, and 4.36 ± 1.74 mA for multimodal responses. There were no significant differences among the stimulation current intensities for the five categories (ANOVA, *p* = 0.09; Supplementary Figure 2).

### A diverse repertoire of responses

The responses obtained and the proportion of each category for each separate nucleus was shown in Supplementary Figure 3. The spatial distribution of contacts within the BG subnuclei and the elicitation rates of different responses/behaviors for each BG subnucleus are illustrated in Fig. 3b.

Of the five response categories, sensorimotor responses (23 sites in 17 patients) were the most frequently observed. Deviation of the eyes and head toward the stimulated side was obtained in 2 sites in the right dCa. Furthermore, a raised contralateral index finger (1 site) and arm (1 site) and a flexed contralateral knee with tremor (1 site) were observed in two patients in response to stimulation in the dlPu. Contralateral tongue muscle contraction and subsequently arrested speech were evoked by stimulation of one site in the left dlPu. Remarkably, complex dystonic gestures that consisted of opening the mouth and slowly spreading all five fingers of the contralateral hand was elicited by stimulating the vmPu. The elicited somatosensory responses were described as pain (1 site in the vmPu and 1 site in the STN) or numbness (10 sites in 11 patients: 4 in the vmPu, 4 in the dlPu, and 2 in the STN) covering the contralateral face, head, limb, and body. Specifically, one patient reported a feeling of electric shock and warmth over the contralateral limb and trunk (one site in the left dlPu). Overall, these sensorimotor reports indicated that the body movement responses were primarily mediated by the contralateral putamen, while the eye movements were associated with the caudate.

Surprisingly, vestibular sensations represented the second most frequent response category, accounting for 8 sites in five patients. A feeling of vertigo was reported as the spinning of the body and/or surroundings (1 site in the vmPu and 2 sites in the dlPu). Three sites (2 in the vmPu and 1 in the dlPu) were detected in two patients who described their experience as follows: “My whole body is very light, like I am floating in the air.” “I feel the left side of my body is light.” Two patients described a sensation of dizziness during stimulation of the vmPu and GPe. Based on the spatial distribution of these sites, vestibular processes predominantly involved the putamen and GPe.

Autonomic responses were also unexpectedly identified at 7 sites in 5 patients. Three patients reported experiencing heart palpitations during stimulation of sites in the putamen (2 in the vmPu and 2 in the dlPu), and two patients reported experiences of nausea when stimulation was applied to sites in the left dCa and vmPu (one site at each location). The responses based on stimulation within the striatum were characterized as heart palpitations and nausea, while responses caused by STN stimulation were described as interoceptive sensations. Regarding complex interoceptive sensations, for example, one patient stated the following after undergoing stimulation in the right STN: “I feel my internal organs are twitching, and a puff of gas flow at the chest level, and with a sensation of little fuzzy pain”.

Distinct cognitive responses were elicited by stimulation of the left vmPu, right dCa, and right GPe (7 sites in five patients). Among these, aphasia without contraction of pharyngolaryngeal muscles was obtained from three stimulation sites located in the left vmPu of two patients. Notably, upon stimulation of one contact located in the left vmPu, one participant reported, “My thoughts are racing out of control.” During stimulation applied to one site within the GPe, one patient stated, “I have the feeling of going into a trance, with blurred vision.” One patient reported experiencing a body schema characterized by his left upper limb and his jaw not belonging to him during stimulation of two sites in the right dCa. Together, these observations support the idea that the left vmPu, right dCa, and right GPe may engage in cognitive processes.

Multimodal responses (nine sites in seven patients), representing a combination of responses from two or more other categories, included autonomic combined with sensorimotor responses (1 site in the dCa, 3 sites in the vmPu, 1 site in the dlPu, and 1 site in the GPe), vestibular sensations plus autonomic responses (1 site in the vmPu), and vestibular sensations co-occurring with sensorimotor responses (1 site in the vmPu). One further patient reported vestibular sensations and autonomic responses mingled with sensorimotor responses when we stimulated one site in the STN.

### The relation of electrophysiological findings to current knowledge of BG function derived from functional magnetic resonance imaging

**Fig. 4:**
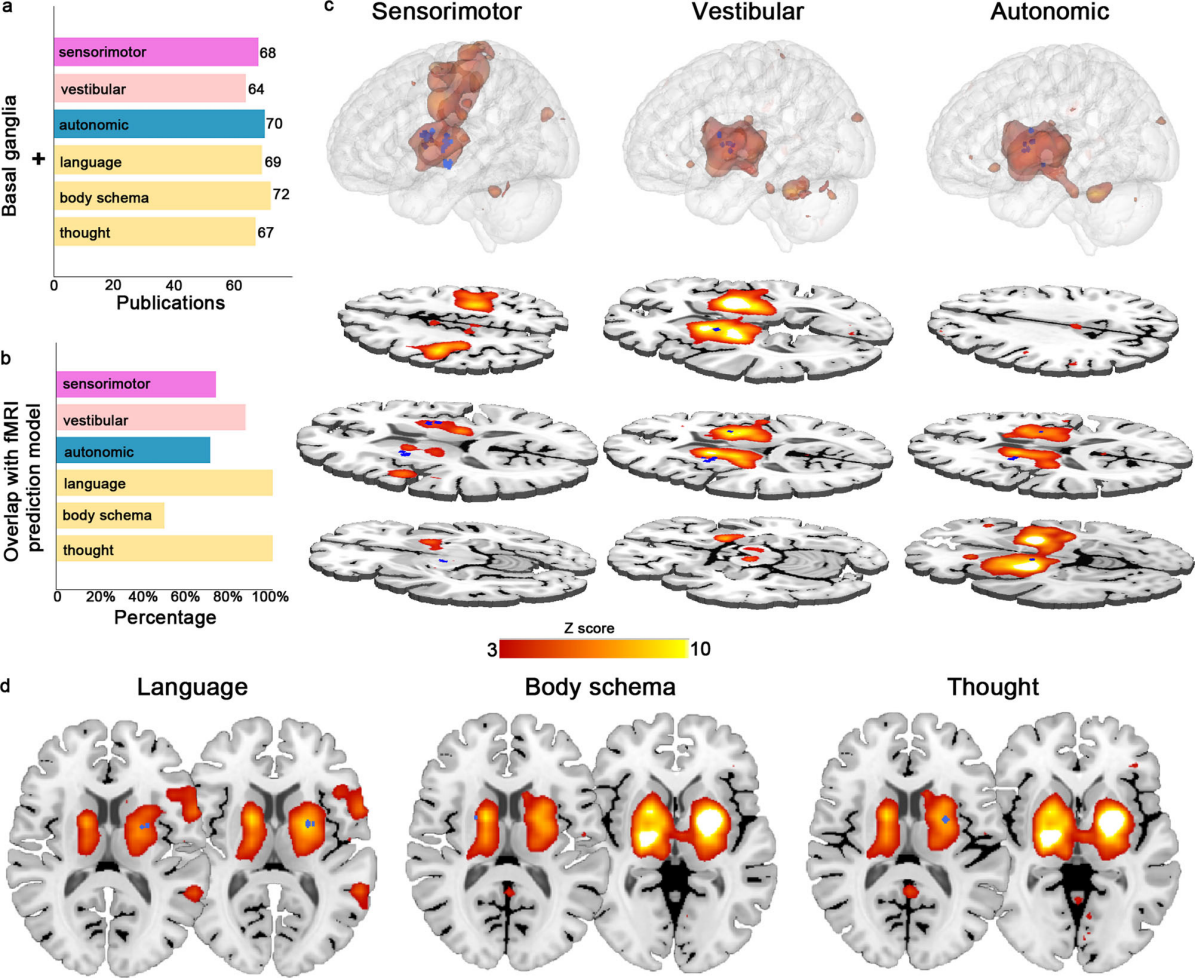
Comparison with the model derived from functional neuroimaging meta-analyses. **a** The numbers of publications obtained from NeuroQuery using queries combining “basal ganglia” with sensorimotor, vestibular, and autonomic response categories and with language, body schema, and thought in the cognitive category. **b** The percentages of overlap between sites at which responses were elicited and the predicted functional map. **c**, **d** Illustrations of each model derived from functional neuroimaging meta-analyses (heatmap) and the locations of the sites at which stimulation elicited the corresponding responses (the blue dots). **c** The relationship between the predicted map and the spatial distribution of the sites at which sensorimotor, vestibular, and autonomic responses were elicited is displayed in 3D and representative slices. **d** Considering the complexity of cognition, within the category of cognitive responses, the sites that elicited language, body schema, and thought when stimulated are displayed separately on the predicted map.

A wealth of functional magnetic resonance imaging (fMRI) studies have implicated the BG in sensorimotor processing as well as more complex affective and cognitive processes. Considering that meta-analysis is a powerful approach to reflect objective evidence in a given field by assessing consistency across the literature and discarding the artifacts, we compared our electrophysiological results with current knowledge of human BG function based on a large amount of functional neuroimaging data. By means of a supervised machine learning model, NeuroQuery (https://neuroquery.org/), a recently described interactive online meta-analysis tool for functional neuroimaging, can produce high-quality mappings to predict the spatial distribution of specific functions in the brain by parsing the relevance of text^27^; NeuroQuery outperforms other tools in quantitative mapping based on studies with a wide range of variation, especially for some concepts that are rarely studied. We searched for the terms “basal ganglia” combined with specific categories of elicited responses (“sensorimotor”; “vestibular”; “autonomic”; and cognitive categories of “language”, “body schema”, “thought”); these searches yielded matching reports of corresponding behaviors from 13,459 full-text publications^24^ (Fig. 4a). From these results, we obtained a predicted brain map associated with each of the six category terms. In general, high levels of overlap were detected between the observed response sites and predicted brain maps (Fig. 4b, c). For example, 73.9% of locations that we observed to elicit sensorimotor responses were located in the brain regions that were predicted to be activated. However, some locations with empirically observed roles in the functional architecture of the BG were not especially predicted. As shown in the middle panel of Fig. 4d, only one of two adjacent contacts that elicited body schema alterations (i.e., a disturbing sensation of limb ownership) was predicted.

## Discussion

To our knowledge, this is the first and currently the most comprehensive exploration of the human BG functional architecture by multisite electrical stimulation with SEEG; the results provide an overview of the physiological responses and the anatomical location. At present, our report is the only original account of these physiologically evoked clinical responses extending not only to the expected array of mostly sensorimotor responses, but also to vestibular sensations, autonomic responses, cognitive effects, and multimodal effects. This report made the possibility to map human BG function by the use of DES in a series of patients with refractory epilepsy who underwent SEEG recordings as part of their presurgical evaluation. Neuroimaging findings have noted the hemispheric dominance of different BG functions, particularly for high-order functions^10^. Though the sparse spatial distribution of BG response sites, we keep the original coordinate of the responding site. The spatial distribution of identified behaviors in the BG largely correlates with the corresponding predicted map derived from large-scale meta-analyses. However, the unpredicted locations in our findings emphasize that some of the functional architecture of the human BG is still outside of the scope of current knowledge.

SEEG, as pioneered by Bancaud and Talairach in 1965, is now used for comprehensive presurgical evaluation of refractory epilepsy. SEEG is indicated for candidates for epilepsy surgery when a surgical hypothesis exists, but anatomo-electro-clinical data collected during the noninvasive phase are insufficiently concordant regarding the supposed localization of the region responsible for seizures and/or its relationship with functional areas^28^. Given the advantage of SEEG in the tridimensional and temporally precise study of epileptogenic networks, it has historically been used to explore subcortical structures since its inception^29^. In recent years, by implantation of SEEG electrodes into subcortical structures including the thalamus and BG, different research groups have explored the role of cortical–subcortical network interactions in seizures^30–33^. In our center, SEEG was recently used to investigate the mechanism of ANT DBS for refractory epilepsy and reveal the role of STN played in motor seizures for the purpose of clinical research, the results of which have been partially reported^23,24^. Based on the presurgical evaluation, personalized SEEG electrodes were implanted to investigate the cortico-subcortical epileptic network, the electrodes targeting the ANT or STN would pass through distinct regions of the BG and the electrodes targeting the insula also extended to BG in these series of epilepsy patients without BG functional deficits.

In this scenario, the BG function is assumed to be normal during the interictal period. Findings from patients with epilepsy have shown ictal discharge can be propagated from the cortex to the subcortical structures including BG and thalamus^32,34,35^, though the BG and thalamus themselves are not capable of generating seizures (so, most possible, it is propagated). Of note, the functional connectivity between BG and cortex estimated by resting state fMRI data is likely to be altered in epileptic patients^36^. However, Bouilleret V et al.^37^ showed that BG occurred with no changes in glucose metabolism by studying 12 temporal lobe epilepsy patients using [18 F] fluorodeoxyglucose PET, suggesting insignificant functional impairment of BG. Together with the characterization of the current epilepsy patient group: (1) lacking BG dysfunctional symptoms; (2) the normal structure of BG shown by MRI; and (3) no interictal epileptic discharges in BG, our present study thereby offered a unique chance to investigate the physiological function of the BG.

DES undoubtedly remains a straightforward and classical method for inferring the function of brain areas since its first use in the late nineteenth century. Each eloquent structure, whatever its actual role in brain function, can be electrically perturbed by DES, which will necessarily induce a functional consequence. This optimal sensitivity explains why DES is currently considered the standard in brain mapping^20^. To date, many key cortical functions, including primary and high-order functions have been discovered through DES^38–40^, which provides an entry gate to the whole network that sustains a function. Although the mechanism is still far from completely understood, the stimulation may evoke its effects antidromically to activate in some way connections that produced the observed effects^41^. In addition, a specific response that we observed might be ascribed to the function of the focal area, but it may also be a combined effect of the focal and area remote structures activated by the stimulation of passing fibers^42^, reflecting the same specific functional network or circuit. Considering the heavily interconnected nature of the BG, and their subcortical position among (and traversed by) fiber bundles, it is likely that the stimulation-activated passing fibers either at the edge of the stimulated nucleus or even within the same nucleus (an axon that reaches one part of the BG may travel through other areas of these nuclei)^43,44^. In this scenario, the evoked summation effects over a large brain volume are hard to predict and we cannot be easily discerned where exactly the observed results were generated. Nevertheless, it is reasonable to assume that perturbation induced by DES anywhere in a given functional network will disrupt the same specific network or circuit of the BG^21^.

The stimulation site that most frequently elicited sensorimotor responses was the dorsolateral sector of the striatum, which is the primary recipient of cortical fibers from the primary motor and somatosensory cortices. Our present observation that body movement responses were primarily mediated by the contralateral putamen, while eye movements were related to the caudate, is in concordance with the well-established model supported by numerous studies^3,10,45^. The identified somatosensory responses are consistent with the increasing number of studies suggesting that the BG serves as a gatekeeper for somatosensory inputs and sustains sensory function through the integration of information from the cortex, the thalamus, and many other sites^46–48^. Given the potential spread of the current in DES, we cannot completely rule out the involvement of the fibers carrying somatosensory information. With the cautious restriction of the activated contacts within the BG and avoidance of the potential effects of the internal capsule, our results support the concept of a potential central origin for somatosensory disturbances; such disturbances are a common feature of PD and other circuit disorders.

Maintaining equilibrium is an essential function in mammals and involves the coordination of infratentorial and supratentorial structures^49^. Despite occasional reports of BG lesions being associated with vestibular symptoms, it has been unclear whether BG is indeed involved in the processing of vestibular signals. Thus, the identification of vestibular responses was striking. Indeed, vestibular dysfunction is present in PD patients but has largely been disregarded^50,51^; however, a neuronal tracer study in rats showed the presence of a disynaptic pathway between the vestibular nucleus and the striatum^52^. Although not exactly the same as the prior report showing dlPu involvement in vestibular signal processing, we obtained vestibular sensation responses to vmPu and GPe stimulation as well as stimulation in the proximity of the dlPu. Our data thereby supports the notion that the right putamen and GPe support the integration of vestibular signals, offering critical insight into the underlying pathology of the decrease in balance and the development of postural instability in PD. Importantly, our evidence directly suggests the existence of a functionally segregated vestibular pathway of the BG.

Most autonomic responses that we observed within the striatum and STN involved cardiovascular and gastrointestinal functions, implying that the BG is involved in autonomic modulation. Clinically, the manifestations of autonomic dysfunction, such as gastrointestinal and cardiac autonomic dysfunction, postural hypotension, and bladder, bowel, and sexual dysfunction, are common in PD patients^36–38^. However, autonomic responses following stimulation of the BG have rarely been reported in humans^53^. The well-known central autonomic network, including the insular cortex, anterior cingulate cortex, hypothalamus, amygdala, periaqueductal gray matter, parabrachial complex, the nucleus of the solitary tract, and ventrolateral medulla, is a set of reciprocally interconnected brain areas that control visceromotor, neuroendocrine, and behavioral responses essential for survival^54,55^. Whether the alterations in autonomic modulation in PD are due to changes in processing in the BG has always been a controversial matter. Experimental data have hinted that the BG could be involved in autonomic activity^56,57^. Our observation supports the notion of a possible autonomic collateral circuit in the BG that is crucial to autonomic deficits in patients with PD.

The identification of cognitive responses is enlightening. Stimulation has allowed three types of cognition-related behaviors to be distinctly observed. The role of the BG in language function has been evident in stroke patients with BG lesions since the 1970s^58,59^. Our finding of speech arrest following stimulation in the left putamen is in line with a previous intraoperative electrical stimulation study^60^. Furthermore, alterations in body schema occurred when the right parietal cortex or right insula was lesioned or stimulated, but the related network has not been well elucidated^61^. Body schema alterations elicited by stimulation in the right dCa suggested that the BG engages in the processing of information related to body awareness, which could account for the pathophysiology of alien limb syndrome—the predominant feature in corticobasal syndrome. Interestingly, the transient confusion of thought resulting from left vmPu stimulation might be assumed to reflect a disruption of decision-making or working-memory processing, which also provides a clue regarding chronic intrusive thoughts in obsessive-compulsive disorders.

The classical functional subdivisions of the BG are topographically organized and have distinct anatomical boundaries. Our response data highlight this functional segregation, but also show spatial overlap between different types of responses^62–64^. The spatial overlap and the observation of mingled behavioral responses support the emerging notion that BG functional subdivisions are globally segregated but locally overlapping. This may be attributable to the sharing of the same cytoarchitecture for functional integration in the BG. With a similar stimulation current intensity to other response categories, multimodal responses may be attributable to the convergent role of a shared anatomical substrate in the integration of multiple physiological functions, although the effect of volume conduction or current spread cannot be strictly excluded.

The limitations of the present study should be noted. First, because the implantation of intracranial electrodes was determined on the basis of clinical needs, there was incomplete and uneven coverage of the BG subnuclei, which constrained the comprehensive topographical mapping of the functional architecture of the BG. Second, the clinical procedure of DES aims to identify the eloquent regions and the epileptogenic areas in presurgical evaluation by evaluating the elementary clinical responses, which might be insufficient to evoke particular emotions and cognitive responses in this setting with short stimulation times (3 s); this also might be the underlying reason that stimulation of some sites did not elicit any response. Additionally, there are unavoidable factors that cultural or age-related experiences flavor the descriptions provided by patients that possibly influence the reliability of the subjective symptoms and the cognitive tasks used would impact the high-order functions mapping. Third, we should take into account that the high-order functions governing human complex behaviors may recruit several processes and would overlap, the functional classification is inherently difficult. The classification scheme we used in the current study is based on the functional organization of BG and the characteristic profiles of the responses. Given the limited sample and classification limitation, we provided the initial description of the patients for further analysis.

Overall, our work is the only currently available stimulation study with the aim to evaluate the neurophysiological perspective of BG distinct regions. In addition, our results pave the way for future studies to dissect the potential corresponding functional pathways. Importantly, our findings provide direct electrophysiological evidence for the role of BG in a family of symptoms, such as postural instability, autonomic dysfunction, cognitive disturbances, body awareness alterations, and speech impairments, that are common in PD and other circuit disorders. Moreover, considering that the encouraging effective results of DBS in neuropsychiatric disorders are not always replicated^18,19^, the identified correlations of neuroanatomical substrates with specific responses, along with investigations obtained from other modalities, to improve our deep understanding of the functional characteristic of the critical hubs in brain circuits. Our study would present the prospect of refining neurosurgical targets and yielding potential symptom-specific therapeutic strategies for brain circuit disorders.

## Methods

### Participants

Data from fifty-six patients with refractory focal epilepsy who underwent SEEG between 2017 and 2021 at Xuanwu Hospital, Capital Medical University, Beijing, China, were retrospectively reviewed. All patients required continuous SEEG recordings to precisely localize the epileptogenic zone or map the eloquent cortex because of insufficient information from comprehensive noninvasive evaluations, including a detailed history, seizure semiology, concurrent video recording, and scalp EEG, MRI and/or positron emission tomography^65,66^. All the patients included in this study met the following criteria: (1) At least one SEEG electrode was extended into the ANT or STN for the purpose of mapping the cortico-subcortical epileptic network, refining the stimulation target, or probing the mechanism of DBS^23,24^. (2) The trajectory of the above electrode passed through the BG. (3) DES was performed as the electrode passed through the BG. (4) There was no significant brain deformation due to lesions or encephalomalacia. (5) No psychiatric comorbidities and other surgical contraindications. Ultimately, 39 patients (19 males, 20 females; age: 14~38 (23.7 ± 6.21) years) were identified with at least one electrode passed through BG (the demographic and clinical characteristics are detailed in Supplementary Table 1).

### Ethics and patient privacy

All patients aged ≥18 years provided informed consent while patients aged 14–17 years authorized their parents to permit them to participate in this human clinical trial and provided consent to participate in the study, which is in accordance with the Ethics Committee of Xuanwu Hospital for human clinical experience. In addition, for patients aged <18 years, at least one parent would be asked to accompany them during the clinical test. Approval for conducting the proposed research was obtained through the Ethics Committee of Xuanwu Hospital, Capital Medical University. To ensure the confidentiality of the participants’ information, all data were deidentified, and arbitrary codes were assigned to each patient’s data.

### SEEG electrode implantation and the reconstruction of depth electrodes

All patients underwent high-resolution T1-weighted MRI scans (3.0 T, Siemens) before surgery and three-dimensional computed tomography (CT) scans within 24 hours after SEEG implantation to verify electrode placement. The SEEG electrodes were implanted using an oblique approach under general anesthesia. The procedure for SEEG electrode implantation has been described in detail in our previous publications^23,24^. The multisite SEEG electrodes were semirigid platinum/iridium, and they were 0.8 mm in diameter, had 8–20 contacts, measured 2 mm in length, and were spaced 1.5 mm apart (Sinovation, China).

Each patient’s neuroimaging data were processed in Lead-DBS (https://www.lead-dbs.org/)^67^, and all SEEG electrode locations were reconstructed in standard MNI space on what is currently the highest-resolution template available (MNI152 ICBM2019b). Briefly, each patient’s postoperative CT scan was first linearly coregistered to the preoperative MRI scan using Statistical Parametric Mapping 12 (https://www.fil.ion.ucl.ac.uk/spm/software/spm12). The registration between postoperative CT (Siemens) and preoperative T1 images was further refined using the ‘brain shift correction’ module in Lead-DBS, which focused on the subcortical target region of interest. Afterward, the data were normalized into MNI152 space with the symmetric diffeomorphic registration algorithm implemented in Advanced Normalization Tools (http://stnava.github.io/ANTs/). The coregistration and normalization were visually verified, adjusted, and refined using the subcortical transformation step. The precise location of each contact in the MNI152 space was manually verified by an experienced user with ITK-SNAP software (http://www.itksnap.org/pmwiki/pmwiki.php). Each SEEG trajectory and all contacts were automatically reconstructed using MATLAB based on the SEEG leads’ deepest points and the base points. The pipeline for SEEG reconstruction is shown in Supplementary Figure 1a.

### SEEG recordings and stimulation procedure

SEEG signals were routinely recorded for 7–14 days via a Micromed video-EEG monitoring system (sampling rate = 1024 Hz; 50-Hz notch filter) to capture at least three habitual clinical seizures after surgery. During chronic SEEG monitoring, high-frequency DES was conducted^23,24,68^. Electrical pulses were generated with Grass S88 (SUI-7, Astro-Med Inc.). All parameters were selected to avoid any tissue injury and to optimize the yield of the stimulation (charge density per square pulse <55 lC per cm^2^)^69,70^. Typical bipolar stimulations of two serial and adjacent contacts were carried out by applying biphasic rectangular stimuli of alternating polarity.

High-frequency stimulation at 50 Hz (pulse width: 0.3 ms; duration: 3 s; charge-balanced biphasic rectangular stimuli of alternating polarity) was applied as a routine part of the standard presurgical assessment for functional mapping. None of the patients had seizures within 2 h prior to functional mapping. During the high-frequency stimulation, the patients were asked to sit on a bed and remain in a resting state. To avoid the possible inhibitory effects of the white matter due to the prior stimulation of the cortex, we routinely stimulate the electrode from the deepest contact to the superficial contact. The patients were blinded to the time points when the stimulation was delivered. In addition, various tasks by asking questions, particularly the language, would be performed during the mapping test. For instance, we would ask the patient to count aloud and read the sentence, naming or repetition, chosen as previously described^71,72^. To avoid a baseline error rate, the tasks were performed before DES and were not interrupted during the time between stimulations. The patients were asked to report any elicited experiences, and any objective responses/behaviors were carefully observed and recorded. For the stimulation session, the current intensity initially ranged from 0.5–1.0 mA and increased by 0.5 mA up to elicit electrical after discharges or behavioral responses observed, but the maximum stimulation intensity was not >8 mA^73^. The obvious responses/behaviors would be observed when the stimulation started, and the patient recovered to rest when the stimulation stopped. There is a strict time-locked relationship between the stimulation and the responses. If any subjective or objective responses were evoked, stimulation was repeated three times with the same parameters to confirm the reliability of the responses (considered “reproducible”)^71,72,74^. In addition, the interval time between each stimulation was not fixed, ~30 s in most cases, depending on the time length the patient came back to the baseline if a certain response was elicited. It might also increase if the testing equipment needed to be adjusted or if the testing person needed to make notes about the responses. Also, if after discharges occurred, the next stimulation wouldn’t exert until they stopped. All responses induced by stimulation were video recorded and stored in clinical report documents with the patient’s descriptions of their experiences.

### Data processing

The elicited responses were first analyzed based on the video–EEG recordings of the stimulation sessions and the patient’s self-reports and/or the observed clinical responses. To avoid false positive results, only the lowest stimulation intensity that elicited a clinical response was included for analysis. In other words, intensities below the analyzed level did not induce any observable responses. To investigate the functional architecture of the BG based on the responses to stimulation, the anatomical locations of the electrode contacts evoking similar responses in MNI152 space were mapped to BG substructures.

The BG were segmented into the following four substructures, as defined by the DISTAL Atlas^25^: (1) the striatum, (2) the GPe, (3) the GPi, and (4) the STN. Next, using the Brainnetome atlas, which provides the subregions of the caudate and putamen, the caudate was further divided into dorsal and ventral parts (dCa and vCa), while the putamen was divided into ventromedial and dorsolateral parts (vmPu and dlPu)^26^. Finally, the BG were separately parcellated into their seven component substructures in each hemisphere (Supplementary Fig. 1b). The electrode contacts localized in these above subsections were automatically identified, visually double-checked, and used for further analysis.

### Comparison with the NeuroQuery-derived model based on fMRI

Assembling evidence across a bulk of studies is crucial to acquire a comprehensive view of specific brain functions between brain and behaviors. The classic meta-analysis methods, such as Coordinate-Based Meta-Analysis^75–77^, Activation Likelihood Estimate^75^, and Multilevel Kernel Density Analysis^76^, which perform statistical tests on a particular term relying on in-sample published studies are hard to control the concepts with contrast description. In contrast to the traditional methods, the recently described automated online meta-analysis tool NeuroQuery predicts the most relevant brain regions by parsing the queries using supervised machine learning from a database derived from 13,459 full-text articles^27^. The brain map produced by NeuroQuery has been shown to predict the spatial distribution of findings well and thus provide a good basis to generate regions of interest or interpret results for studies of rarely examined terms^78,79^. To generate good prediction maps in NeuroQuery, we used combinations of the term “basal ganglia” and the term for specific categories of elicited responses (“sensorimotor”; “vestibular”; “autonomic”). Since the distinct property, “language”, “body schema”, and “thought” of cognitive responses were separately examined. The predicted brain maps were thresholded using clusters determined by Z > 3 (voxelwise one-tailed *p* < 0.001), for instance, in the selection of typical regions for querying^27^. Next, to compare the spatial distribution of the elicited responses with the resulting whole-brain maps from NeuroQuery, we created a four-millimeter spherical region of interest centered at each stimulation site in the template space (MNI152) that elicited responses. We then overlayed the region of interest on the predicted brain maps for comparison.

### Statistics and reproducibility

Data visualization was performed using Lead-DBS and FSL (https://fsl.fmrib.ox.ac.uk/fsl/fslwiki). For statistical analysis, following ANOVA, multiple two-sample *t* tests were used to compare the intensity of delivered to responsive and nonresponsive within the BG; a correction was applied for multiple comparisons. The statistical significance level was set at *p* < 0.05 (Supplementary Figure 3). Results were collected from the patients whose SEEG electrode was strictly located in BG and recorded no interictal discharges (*n* = 35). For any subjective or objective responses that were evoked, stimulation was repeated three times with the same parameters to confirm the reliability of the responses (considered as “reproducible”).

### Reporting summary

Further information on research design is available in the Nature Research Reporting Summary linked to this article.

## Supplementary information

Supplementary Information (new)

Description of Additional Supplementary Files

Supplementary Data 1

Supplementary Data 2

Reporting Summary

## Data Availability

The data that support the findings in this report are available in the report itself and in the Supplementary information, and source data underlying Fig. 4a, b and provided in Supplementary Data 2. In the interest of maintaining the patient’s privacy, the raw data are not publicly available due to the privacy of the patients but are available from the corresponding author upon reasonable request.

## Code availability

The code used for data processing, electrode reconstruction, and data visualization is openly available from public toolboxes (https://www.leaddbs.org; https://fsl.fmrib.ox.ac.uk/fsl/fslwiki; https://www.fil.ion.ucl.ac.uk/spm/software/spm12; http://stnava.github.io/ANTs; https://neuroquery.org).
