# Supplementary material for: Intracranial direct electrical mapping reveals the functional architecture of the human basal ganglia
Source: Commun Biol. 2022 Oct 23;5:1123. doi: 10.1038/s42003-022-04084-3 (PMC9588773; doi:10.1038/s42003-022-04084-3)
Supplement: Supplementary file 1 — Supplementary Information (new) [file 42003_2022_4084_MOESM1_ESM.pdf]

Supplementary information

Intracranial direct electrical mapping reveals the functional architecture of the human basal ganglia

Lei Qi, Cuiping Xu, Xueyuan Wang, Jialin Du, Quansheng He, Di Wu, Xiaopeng Wang, Guangyuan Jin, Qiao Wang, Jia Chen, Di Wang, Huaqiang Zhang, Xiaohua Zhang, Penghu Wei, Yongzhi Shan, Zaixu Cui, Yuping Wang, Yousheng Shu, Guoguang Zhao, Tao Yu, Liankun Ren

Supplementary Table 1 Clinical characteristics of the patients whose electrode by pass basal ganglia

PatNum	Gender	Age	Dominate_ hand	Seizure Type	SEEG _side	SEE G_N um	BGE LE_ Num	ELE_BG_ Target	Psychiatric diagnoses	Medicine
P1	Male	23	Right	FAS; FBTCS	Right	6	1	ANT	Normal	VPA+LEV+OX C+LTG
P2	Male	18	Right	FAS; FBTCS	Bilater al	8	1	ANT	Normal	VPA+LTG
P3	Male	24	Right	FIAS; FBTCS	Right	7	1	ANT	Normal	VPA+PB
P4	Female	23	Right	FAS; FBTCS	Right	7	1	ANT	Normal	OXC+LEV
P5	Female	26	Right	FIAS	Right	6	1	ANT	Normal	OXC+LEV
p6	Male	30	Right	FIAS; FBTCS	Bilater al	7	2	ANT& insula	Normal	OXC+LTG+TP M
p7	Female	32	Right	FIAS	Bilater al	5	1	ANT	Normal	LEV+LTG
p8	Female	18	Right	FAS; FBTCS	Right	6	1	ANT	Normal	PHT
p9	Male	26	Right	FIAS; FBTCS	Right	6	1	ANT	Normal	VPA+LTG+LE V
P10	Female	16	Right	FIAS; FBTCS	Right	6	2	ANT & insula	Normal	LEV+OXC
P11	Female	16	Right	FAS; FBTCS	Bilater al	7	1	ANT	Normal	CBZ+LTG+LE V+TPM+CZP
P12	Male	24	Right	FAS; FBTCS	Bilater al	6	1	ANT	Normal	LEV+VPA+TP M+CBZ
P13	Female	19	Right	FAS; FBTCS	Left	5	1	ANT	Normal	OXC+TPM
P14	Male	27	Right	FAS	Left	7	1	ANT	Normal	OXC
P15	Female	32	Right	FAS	Left	5	1	ANT	Normal	CBZ
P16	Female	20	Right	FAS; FBTCS	Left	6	1	ANT	Normal	VPA+CBZ+LE V
P17	Female	38	Right	FAS; FBTCS	Right	7	1	ANT	Normal	OXC+LEV+VP A
P18	Female	24	Right	FAS; FNMS	Left	7	1	ANT	Normal	OXC+LEV+VP A
P19	Male	21	Right	FMS; FBTCS	Left	8	1	ANT	Normal	LTG+CBZ+RF N
P20	Female	22	Right	FAS; FBTCS	Right	7	1	ANT	Normal	LEV+OXC
P21	Female	32	Right	FAS	Right	7	1	ANT	Normal	OXC
P22	Female	18	Right	FIAS; FBTCS	Right	8	1	ANT	Normal	VPA+TPM
P23	Female	35	Right	FAS; FNMS	Right	7	1	ANT	Normal	OXC+LEV
P24	Male	18	Right	FAS; FNMS	Right	6	1	ANT	Normal	LEV+OXC+LT G
P25	Female	20	Right	FAS; FBTCS	Left	7	1	ANT	Normal	OXC+LEV
P26	Male	25	Right	FAS; FNMS	Left	7	1	ANT	Normal	VPA+LEV
P27	Male	15	Right	FAS; FNMS	Right	8	1	ANT	Normal	OXC
P28	Male	28	Right	FAS; FBTCS	Bilater al	7	1	ANT	Normal	CBZ+VPA
P29	Male	31	Right	FAS; FNMS	Left	9	1	ANT	Normal	OXC+VPA
P30	Male	17	Right	FIAS	Bilater al	8	1	ANT	Normal	OXC+TPM
P31	Female	16	Right	FAS; FNMS	Bilater al	9	1	ANT	Normal	LTG

P32	Female	28	Right	FAS; FBTCS	Bilateral	7	1	ANT	Normal	LTG+TPM
P33	Male	31	Right	FMS	Left	7	1	STN	Normal	PHT+VPA+LTG+OXC
P34	Female	21	Right	FMS; FBTCS	Bilateral	10	1	STN	Normal	LEV+PHT
P35	Male	18	Right	FMS	Left	6	2	STN	Normal	TPM+CBZ
P36	Male	29	Right	FMS	Left	7	1	STN	Normal	VPA+TPM
P37	Male	19	Right	FMS	Left	8	1	STN	Normal	VPA+ZNS
P38	Male	14	Right	FMS; FBTCS	Bilateral	13	1	STN	Normal	VPA+TPM
P39	Female	33	Right	FMS; FBTCS	Bilateral	8	1	STN & & insula	Normal	LEV+OXC

ANT: anterior nucleus of thalamus; BG: basal ganglia; CBZ: Carbamazepine; CZP: Clonazepam; EP: epilepsy; ELE: electrode; FAS: focal aware seizure; FBTCS: focal to bilateral tonic-clonic seizure; FMS: focal motor seizure; FNMS: focal nonmotor seizure; FIAS: focal impaired awareness seizure; LEV: Levetirancetam; LTG: Lamotrigine; Num: numbers; OXC: Oxcarbazepine; Pat: patient; PB: Phenobarbital; PHT: Phenytoin; RFN: Rufinamide; STN: subthalamic nucleus; TPM: Topiramate; VPA: Valproate; ZNS: Zonisamide.

Supplementary Figure 1: **Pipeline for SEEG reconstruction in MNI standard space and parcellation of BG subnuclei.**

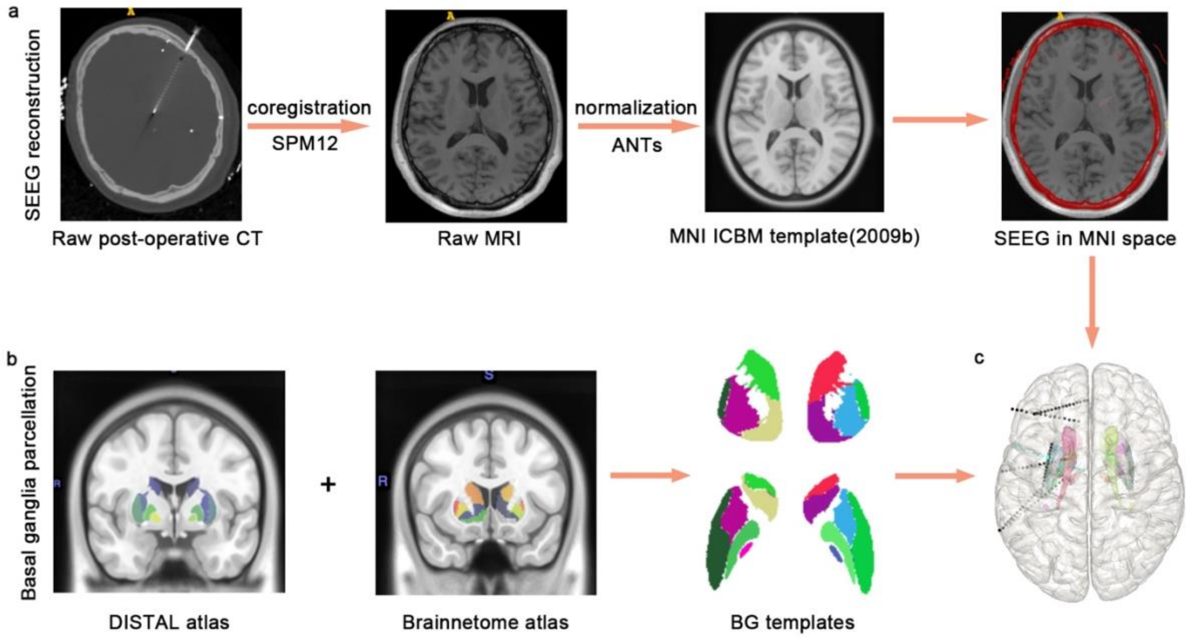

a SEEG reconstruction for an example patient. The postoperative CT was coregistered with the preoperative MRI using SPM12 and normalized to MNI space using ANTs. Next, the implanted multisite SEEG electrodes were reconstructed in MNI space. **b** Pipeline of BG parcellation. The striatum was further parcellated into the dorsal caudate, ventral caudate, ventromedial putamen, and dorsolateral putamen according to the DISTAL and Brainnetome atlases. **c** The SEEG electrodes and the BG parcellation were rendered on a semitransparent 3D brain model in MNI space. SPM, Statistical Parametric Mapping; ANT, Advanced Normalization Tools.

Supplementary Figure 2: Comparison of stimulation current intensity.

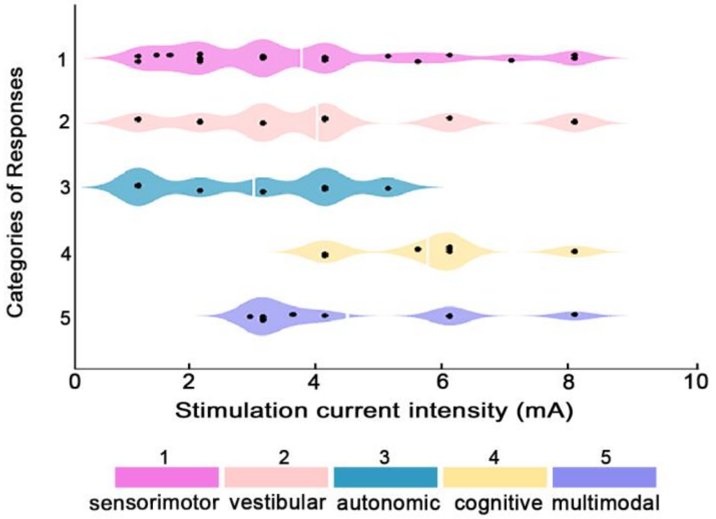

Comparison of stimulation current intensity that elicited responses in each category (ANOVA, p=0.09). The response categories are color-coded.

Supplementary Figure 3: The responses obtained and the proportion of each category for each separate nucleus.

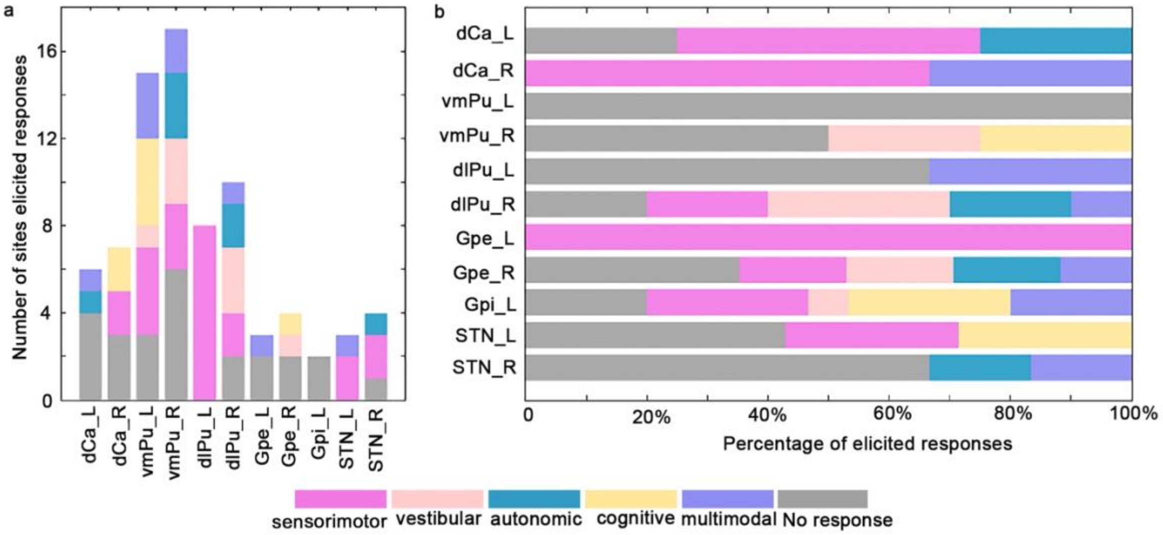

a Absolute numbers of sites eliciting the corresponding responses within each substructure. **b** The percentage of each category of response elicited within each substructure. Different colors correspon to different response categories. L: left; R: right; dCa: dorsal caudate; vmPu: ventromedial putamen; dlPu: dorsolateral puteman; Gpe: external globus pallidus; Gpi: internal globus pallidus; STN: subthalamic nucleus.
