# Supplementary material for: Intracranial direct electrical mapping reveals the functional architecture of the human basal ganglia
Source: Commun Biol. 2022 Oct 23;5:1123. doi: 10.1038/s42003-022-04084-3 (PMC9588773; doi:10.1038/s42003-022-04084-3)
Supplement: Supplementary file 2 — Description of Additional Supplementary Files [file 42003_2022_4084_MOESM2_ESM.pdf]

Description of Additional Supplementary Files

File name: Supplementary Data 1
Description: BG elicited responses and stimulation parameters**.** BG, basal ganglia; dCa, dorsal caudate; dlPu, dorsolateral putamen; GPe, external globus pallidus; GPi, internal globus pallidus; vmPu, ventromedial putamen; STN, subthalamic nucleus; L: left; R: right.

File name: Supplementary Data 2
Description: Source data underlying Figure 4a-b
