# Supplementary material for: Intracranial direct electrical mapping reveals the functional architecture of the human basal ganglia
Source: Commun Biol. 2022 Oct 23;5:1123. doi: 10.1038/s42003-022-04084-3 (PMC9588773; doi:10.1038/s42003-022-04084-3)
Supplement: Supplementary file 5 — Reporting Summary [file 42003_2022_4084_MOESM5_ESM.pdf]

nature portfolio

Corresponding author(s): Liankun Ren

Last updated by author(s): Sep 23, 2022

Reporting Summary

Nature Portfolio wishes to improve the reproducibility of the work that we publish. This form provides structure for consistency and transparency in reporting. For further information on Nature Portfolio policies, see our Editorial Policies and the Editorial Policy Checklist.

Statistics

For all statistical analyses, confirm that the following items are present in the figure legend, table legend, main text, or Methods section.

n/a	Confirmed	
☐	☒	The exact sample size (*n*) for each experimental group/condition, given as a discrete number and unit of measurement
☐	☒	A statement on whether measurements were taken from distinct samples or whether the same sample was measured repeatedly
☐	☒	The statistical test(s) used AND whether they are one- or two-sided<br>*Only common tests should be described solely by name; describe more complex techniques in the Methods section.*
☒	☐	A description of all covariates tested
☒	☐	A description of any assumptions or corrections, such as tests of normality and adjustment for multiple comparisons
☐	☒	A full description of the statistical parameters including central tendency (e.g. means) or other basic estimates (e.g. regression coefficient) AND variation (e.g. standard deviation) or associated estimates of uncertainty (e.g. confidence intervals)
☐	☒	For null hypothesis testing, the test statistic (e.g. *F*, *t*, *r*) with confidence intervals, effect sizes, degrees of freedom and *P* value noted<br>*Give P values as exact values whenever suitable.*
☒	☐	For Bayesian analysis, information on the choice of priors and Markov chain Monte Carlo settings
☒	☐	For hierarchical and complex designs, identification of the appropriate level for tests and full reporting of outcomes
☒	☐	Estimates of effect sizes (e.g. Cohen's *d*, Pearson's *r*), indicating how they were calculated

Our web collection on statistics for biologists contains articles on many of the points above.

Software and code

Policy information about availability of computer code

Data collection	No software was used.
Data analysis	Data analysis was performed using custom code and public toolbox in Matlab as described in the manuscript.

For manuscripts utilizing custom algorithms or software that are central to the research but not yet described in published literature, software must be made available to editors and reviewers. We strongly encourage code deposition in a community repository (e.g. GitHub). See the Nature Portfolio guidelines for submitting code & software for further information.

Data

Policy information about availability of data

All manuscripts must include a data availability statement. This statement should provide the following information, where applicable:

- Accession codes, unique identifiers, or web links for publicly available datasets
- A description of any restrictions on data availability
- For clinical datasets or third party data, please ensure that the statement adheres to our policy

The data that support the findings in this report are available in the report itself and in the Supplementary Information. The source data are not publicly available due to the privacy of the patients, but are available from the corresponding author on reasonable request.

Human research participants

Policy information about studies involving human research participants and Sex and Gender in Research.

Reporting on sex and gender	Male: 19, Female: 20
Population characteristics	This study primarily consist of 39 patients (male: 19, female: 20; age: 14~38 (23.77± 6.21) years old, all right handed) who were diagnosed as refractory epilepsy. Ultimately, 35 patients were included.
Recruitment	Subjects with refractory focal epilepsy who underwent SEEG between 2017 and 2021 were selected based on the following requirements: the patients required continuous SEEG recordings to precisely localize the epileptogenic zone or map the eloquent cortex because of insufficient information from comprehensive noninvasive evaluations; at least one SEEG electrode was extended into the thalamus for the purpose of mapping the cortico-subcortical epileptic network, refining the stimulation target, or probing the mechanism of deep brain stimulation; the trajectory of the above electrode passed through the basal ganglia; direct electrical stimulation was performed as the electrode passed through the basal ganglia; there was no significant brain deformation due to lesions or encephalomalacia.
Ethics oversight	All patients aged ≥ 18 years provided informed consent while patients aged 14-17 years authorized their parents to permit them to participate in this human clinical trial and provided consent to participate in the study, which is in accordance with the Ethics Committee of Xuanwu Hospital for human clinical experience. In addition, for patients aged<18 years, at least one parent would be asked to accompany them during the clinical test. Approval for conducting the proposed research was obtained through the Ethics Committee of Xuanwu Hospital, Capital Medical University.

Note that full information on the approval of the study protocol must also be provided in the manuscript.

Field-specific reporting

Please select the one below that is the best fit for your research. If you are not sure, read the appropriate sections before making your selection.

☒ Life sciences ☐ Behavioural & social sciences ☐ Ecological, evolutionary & environmental sciences

For a reference copy of the document with all sections, see nature.com/documents/nr-reporting-summary-flat.pdf

Life sciences study design

All studies must disclose on these points even when the disclosure is negative.

Sample size	There is no standard method for estimating sample size for this type of study. We attempt to identify as many dataset as possible to map the functional architecture of basal ganglia. To our knowledge, this is the first and largest study of direct intracranial basal ganglia mapping.
Data exclusions	To avoid the potential effects of stimulating the adjacent fibers, we initially took care to exclude the patients whose contacts appeared to cross the boundary of the BG by visual inspection. In addition, stimulation eliciting electrical afterdischarge in basal ganglia was discarded.
Replication	As described in the manuscript, we replicated the stimulation with the same parameters three times when patient reported responses to ensure the findings are consistent.
Randomization	The data is not randomized because the electrode implantation is precisely planned for clinical diagnose and treatment.
Blinding	The patients were blinded to the stimulation time points. Investigators were not blinded.

Reporting for specific materials, systems and methods

We require information from authors about some types of materials, experimental systems and methods used in many studies. Here, indicate whether each material, system or method listed is relevant to your study. If you are not sure if a list item applies to your research, read the appropriate section before selecting a response.

Materials & experimental systems

n/a	Involved in the study
☒	☐ Antibodies
☒	☐ Eukaryotic cell lines
☒	☐ Palaeontology and archaeology
☒	☐ Animals and other organisms
☒	☐ Clinical data
☒	☐ Dual use research of concern

Methods

n/a	Involved in the study
☒	☐ ChIP-seq
☒	☐ Flow cytometry
☒	☐ MRI-based neuroimaging
